# PARP-1: Friend or Foe of DNA Damage and Repair in Tumorigenesis?

**DOI:** 10.3390/cancers5030943

**Published:** 2013-07-26

**Authors:** Amanda F. Swindall, Jennifer A. Stanley, Eddy S. Yang

**Affiliations:** 1 Department of Radiation Oncology Comprehensive Cancer Center, University of Alabama at Birmingham School of Medicine, 176F HSROC Suite 2232B, 1700 6th Avenue South, Birmingham, AL 35249, USA; E-Mails: swindall@uab.edu (A.F.S.); jenn87@uab.edu (J.A.S.); 2 Department of Cell, Developmental and Integrative Biology, University of Alabama at Birmingham, Birmingham, AL 35249, USA; 3 Department of Pharmacology and Toxicology, University of Alabama at Birmingham, Birmingham, AL 35249, USA

**Keywords:** PARP-1, oxidative clustered DNA lesions, inflammation, NFκB, PARP inhibitor, reactive oxygen species, ROS

## Abstract

Oxidative stress induced by reactive oxygen species can result in DNA damage within cells and subsequently increase risk for carcinogenesis. This may be averted by repair of DNA damage through the base or nucleotide excision repair (BER/NER) pathways. PARP, a BER protein, is known for its role in DNA-repair. However, multiple lesions can occur within a small range of DNA, known as oxidative clustered DNA lesions (OCDLs), which are difficult to repair and may lead to the more severe DNA double-strand break (DSB). Inefficient DSB repair can then result in increased mutagenesis and neoplastic transformation. OCDLs occur more frequently within a variety of tumor tissues. Interestingly, PARP is highly expressed in several human cancers. Additionally, chronic inflammation may contribute to tumorigenesis through ROS-induced DNA damage. Furthermore, PARP can modulate inflammation through interaction with NFκB and regulating the expression of inflammatory signaling molecules. Thus, the upregulation of PARP may present a double-edged sword. PARP is needed to repair ROS-induced DNA lesions, but PARP expression may lead to increased inflammation via upregulation of NFκB signaling. Here, we discuss the role of PARP in the repair of oxidative damage *versus* the formation of OCDLs and speculate on the feasibility of PARP inhibition for the treatment and prevention of cancers by exploiting its role in inflammation.

## 1. Introduction

Human tissues are exposed to various sources of reactive oxygen species, which can lead to DNA damage including single strand breaks (SSBs) and the more severe, double strand breaks (DSBs). Reactive oxygen species (ROS) are oxygen derivatives that are highly volatile toward cellular components. Sources of the damaging ROS can be both exogenous (UV light, pollution, ionizing radiation) and endogenous (cellular metabolism and inflammation) [1,2]. Cellular homeostasis depends on a delicate balance between damage *versus* the repair of ROS-induced lesions that occur in lipids, proteins, and DNA. While cellular processes allow for the turnover of lipids and proteins damaged by interaction with ROS, DNA is non-recyclable, and therefore DNA damages must be repaired. Thus, cells have adapted elaborate processes to address DNA lesions caused by ROS, and these lesions are usually fixed by base excision repair (BER) and nucleotide excision repair (NER) pathways upon activation of cell cycle checkpoints [3]. However, ineffective attempts at these processes due to repair pathway deficiencies or clustered DNA lesions can result in cytotoxic or mutagenic effects, and chromosomal instability, all of which may contribute to tumorigenesis [4].

In this review, we examine the possibility of the double-edged sword of the key BER protein PARP-1 (poly-ADP-ribose polymerase) in tumorigenesis by considering its role in ROS-induced DNA damage repair *versus* its role in inflammation. On one hand, PARP1 upregulation may lead to increased DNA repair of ROS-mediated DNA damage. However, continued activation of PARP-1, especially in situations of chronic inflammation/oxidation, may contribute to the formation of OCDLs and enhance tumorigenesis. Given these two diverging roles of PARP-1, the PARP inhibitors may be used to not only treat cancers but also to prevent cancers that form from the potentiation of inflammation/ROS-induced damage caused by persistent PARP-1 upregulation in response to DNA damage. Thus, we will discuss the possibility of using PARP inhibitors in the treatment of not only familial cancers, but also as part of a combination therapy or preventative reagent in non-familial cancers, and potentially in inflammatory diseases which carry a high risk for cancer development.

## 2. PARP-1

PARP-1 (also known as ADP-ribosyltransferase-1, or ARTD1) is a member of the poly-ADP-ribose polymerase family, which is a group of 18 enzymes shown to be involved in many processes ranging from DNA repair to cell death [5]. The most well-studied family member, PARP-1, contains three domains including: (1) zinc-finger DNA binding domain; (2) an automodification domain; and (3) a catalytic domain [6]. The catalytic domain is responsible for the enzymatic activity of the protein. This domain functions to catalyze the addition of ADP-ribose chains to target proteins, including itself, from NAD^+^ donor molecules, resulting in polymer strands in a process known as PARylation. While the PARP family of enzymes is highly conserved, and all contain a catalytic domain signature motif, not all PARP family members have confirmed PARylation activity to date [5]. They are classified as PARP members due solely to the presence of the signature catalytic motif. This review will focus solely on the role of PARP-1.

PARP-1 has been shown to play a role in several nuclear processes including chromatin modification, transcriptional regulation, and DNA damage repair [7]. PARP-1 binds to DNA not only at sites of damage (SSB and DSB), but also DNA crossovers, supercoils and cruciform [8]. The role of PARP-1 in chromatin modification is multifaceted, as studies have demonstrated that PARP-1 can both relax and condense chromatin through interaction with nucleosomes and PARylation of proteins involved in chromatin structure, such as histones H1 and H2B [9,10,11]. Additionally, PARP-1 can regulate transcription through direct interaction with transcription factors, as well as altering their activity via PARylation. For instance, PARP-1 regulates Cox-2, Oct1, E2F-1, and Ap2 through direct interaction, while p53 and RNA polymerases I and II are regulated by PARylation [12,13,14,15,16,17,18]. Additionally, both direct interaction and enzymatic activity by PARP-1 can alter the function of transcription factors, as is the case for NFκB, where PARP-1 binds to and PARylates both p50 and p65 subunits of NFκB [19,20]. The role of PARP-1, either to enhance or repress function of the targets, in each case is unique to the transcription factor and expression level of PARP-1 at a given time.

Perhaps one of the most well known functions of PARP-1 is the role it plays in the sensing and initiation of DNA repair. PARP-1 has been demonstrated to play a role in most forms of DNA repair; including single strand break (base excision repair-BER) and double strand break (homologous recombination [HR] and non-homologous end joining [NHEJ] repair processes [21].

The mechanisms by which PARP-1 contributes to HR and NHEJ are not as well defined as the role in BER/SSB repair. DSB are sensed by the Mre11-Rad50-Nbs1 complex, which binds to the lesion site on the DNA. Subsequently, ATM is phosphorylated and the repair pathway is initiated [22]. The repair pathway choice is determined by cell cycle stage, structure of the DNA break, and availability of homologous DNA [23]. It has been shown that PARP-1 interacts with several proteins involved in the DSB repair pathways including NBS1, Mre11, Ku80, DNA-PKcs and ATM [24,25,26,27]. More specifically, knockdown and chemical inhibition of PARP-1 revealed the interaction between ATM and PARP-1 is important in the kinetics of phosphorylation of downstream signaling molecules including p53 and H2AX, where inhibition of PARP-1 delays ATM activity [27]. Additionally, it has been shown that PARP-1 and PARP-2 may detect and recruit the protein Mre11, initiating the repair of stalled replication forks upon treatment with hydroxyurea [28]. Furthermore, PARP^−/−^/ATM^−/−^ and PARP^−/−^/Ku80^−/−^ mice display embryonic lethality associated with genomic instability [29,30]. While specific mechanisms are not yet elucidated, together these studies suggest PARP-1 is an integral player in the DSB repair pathways.

The role of PARP-1 in the repair of SSB/BER is better defined; however, the exact details are still being investigated. BER and SSB repair serve to correct small lesions that are acquired through a variety of insults including ROS damage or subjection to methylating reagents and irradiation [3]. In order to repair these lesions, the cell first transforms the lesion to a single strand break intermediate via the action of DNA glycosylases and APE1 [31]. This SSB intermediate then recruits PARP-1 to the damage site. Enzymatic activity of PARP-1 works to PARylate itself and other substrates to further recruit repair proteins to the breakage site. Several proteins involved in the BER/SSB repair process have been identified to interact with PARP-1 including XRCC1, DNA polymerase β, PCNA and others [31,32,33,34]. Initially PARP-1 was identified as having a role in BER when PARP-1^−/−^ mice demonstrated increased sensitivity to ionizing radiation and oxidative damages, and cells derived from these mice were hypersensitive to alkylating reagents [35]. Furthermore, BER deficiency due to PARP-1 knockdown was more specifically shown when these cells demonstrated difficulty in DNA strand break sealing after treatment with the alkylating agent methylmethanesulfonate [36]. Additionally, other work has shown that PARP-1 deficiency decreases short and long-patch repair of abasic sites [37]. Interestingly, the role of PARP-1 in ROS-induced damage may be countered by involvement in inflammatory signaling. This then presents a double-edged sword of PARP-1 signaling, where it is participating in DNA repair, but may also induce DNA damage through inflammation if chronically activated for repair processes.

### 2.1. PARP-1 in Inflammation

While most well known for its role in base excision repair, PARP-1 also serves to regulate other signaling pathways, such as cell death and inflammation. PARP-1 inhibition has been shown to be beneficial in a milieu of animal models of inflammatory diseases, such as diabetes, asthma and atherosclerosis [38]. The role of PARP-1 in inflammatory signaling is becoming more evident as the focus of study shifts from solely examining its involvement in DNA repair to other processes including mechanisms of inflammation.

PARP has been shown to contribute to inflammatory processes through a variety of means, including the regulation of transcription factors, cytokines, adhesion factors and inflammatory mediators [39,40]. The well-known and thoroughly described transcription factor, NFκB was one of the first mediators of inflammation to be identified as a target for PARP activity [41]. NFκB has been shown to be a binding partner and also to be PARylated by PARP-1, as previously discussed. PARP-1 is known to be a co-activator of NFκB [41,42]. These studies established in PARP^−/−^ mice and cell lines, that NFκB activity was greatly abrogated independent of upstream activation of NFκB [41,42]. PARP^−/−^ mice were protected from LPS-induced endotoxic shock, due to a strong downregulation of downstream signaling components that propagate the inflammatory signal, including inducible nitric oxide synthase (iNOS) and TNFα [42]. Recently, this has been further confirmed as it has been shown that systemic inflammation serves to upregulate several members of the PARP family in mouse hippocampus, including PARP-1,-3, -9, -12 and -14 [43]. Additionally, the levels of PARylation were increased, and importantly, levels of iNOS were upregulated. PARP inhibitor served to protect cells against increased PARylation and increased iNOS gene expression which is an integral player in ROS/NOS-induced stress in systemic inflammatory response [43]. In both of these cases, knockout and inhibition of PARP served to downregulate the inflammatory response by abrogating NFκB signaling.

The role of PARP-induced inflammation is also multifaceted. It serves as a co-factor for NFκB, and also serves to switch cell death pathways from the well-controlled and highly regulated apoptosis to the more inflammatory necrosis in the event of high levels of ROS species [44,45,46]. In the inflammatory response, the cellular environment is subjected to increased DNA insults carried out by inflammation-induced ROS. This then can lead to the upregulation of PARP-1; as expression of PARP-1 is dramatically increased with DNA strand breaks [47]. The upregulation of PARP-1, and subsequent elevated PARP-1 activity, can deplete cellular levels of ATP and NAD, forcing the cell into necrosis [44,45]. While BER/SSB processes are upregulated, the increase in PARP-1 will also allow PARP-1 to serve as a co-activator of NFκB. If the delicate balance of insult-repair ratio is disturbed, rapid energy depletion and necrotic cell death can occur, resulting in increased inflammatory signaling [45]. By serving as both an NFκB coactivator and pushing ROS-exposed cells to necrosis, PARP-1 functions to further propagate the inflammatory signal. Combined with the regulation of cytokines and inflammatory mediators such as iNOS, it is clear that PARP-1 is a potentially substantial contributor to inflammation.

**Figure 1:**
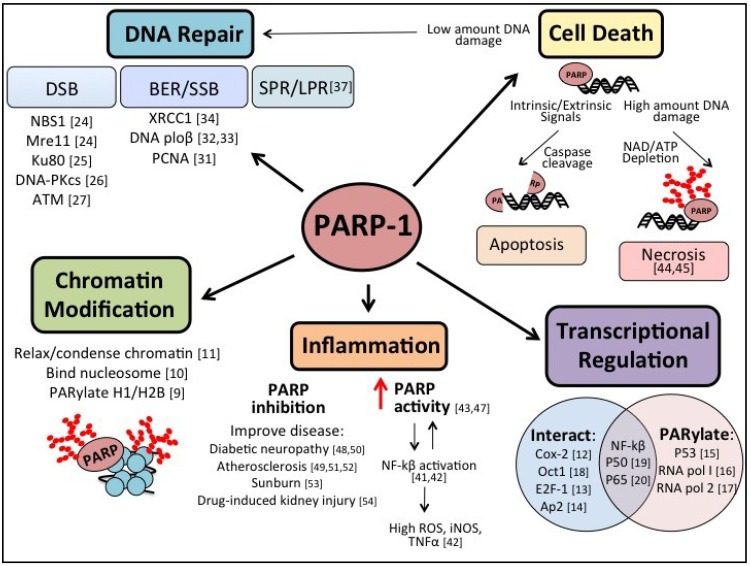
Schematic delineating the multifaceted nature of Poly(ADP) Ribose Polymerase (PARP): DNA repair, Chromatin Modification, Inflammation, Transcriptional Regulation, and Cell Death.

In support of the pro-inflammatory functions of PARP-1, PARP inhibitors have been demonstrated to be an effective treatment of many inflammatory-related diseases in preclinical models. The wide array of inflammatory conditions that have shown improvement by PARP inhibition ranges from chronic diseases such as diabetic neuropathy and atherosclerosis to more acute events such as sunburn-related inflammation and cisplatin-induced kidney inflammation [48,49,50,51,52,53,54]. These inflammatory states are wide in breadth and demonstrate that PARP may contribute to several levels of regulation in a wide range of inflammatory processes and stimuli. These mechanisms are yet to be fully determined and provide subject for further investigation. An overview of the multiple cellular processes regulated by PARP-1 is depicted in Figure 1.

### 2.2. PARP-1 in Cancer

In the updated version of the landmark description of the hallmarks of cancer, Hanahan and Weinberg describe two “enabling characteristics” that allow acquisition of the traditional cancer hallmarks such as resisting cell death and inducing angiogenesis. These enabling characteristics are: (1) genome instability and mutation and (2) tumor-promoting inflammation [55]. Interestingly, PARP-1 sits at the nexus of both of these enabling characteristics. As previously discussed, PARP-1 plays a large role in genome maintenance (DNA repair, chromatin remodeling, transcription factor regulation), and also contributes to the propagation of the inflammatory phenotype. Clinically, the focus of PARP-1 is as a target for the treatment of familial cancers, such as BRCA1/2 deficient breast and ovarian tumors [56]. However, based on its many functions, PARP inhibition may be applicable as a mode of therapy beyond familial cancers.

The majority of the literature to this point has not addressed the possibility of PARP as a pro-tumorigenic factor. This interesting paradigm may be related to its interaction with NFκB, its role in inflammation and subsequent ROS generation, or its pro-survival functions. Mice deficient in PARP have shown decreased susceptibility to skin cancer due to reduced NFκB signaling [57]. In an *in vitro* model of melanoma, PARP-1 driven NFκB signaling led to an increase in the secretion of pro-metastatic cytokines during senescence, termed the PARP-1 NFκB-Associated Secretome, or PNAS. Inhibition of PARP or NFκB in this system abrogates the proinvasive phenotype conferred by PARP-1/NFκB signaling [58]. The extracellular signal-activated kinase, or Erk, has been shown to modulate PARP-1 activity, including the phosphorylation of PARP-1 and the PARylation of NFκB (p65) in an inflammatory response model. This suggests that Erk may also regulate the PARP-1-dependent activation of NFκB [40], further points to a role for this interaction in cancer, as dysregulated Erk pathways are thought to be a present in an estimated one-third of cancers [59]. Additionally, in the mouse model where NFκB signaling was disrupted, a reduction in mammary tumor formation was observed, indicating NFκB signaling as a requirement for tumorigenesis for HER2+ tumors [60]. Interestingly, our laboratory has also shown HER2+ breast cancer cells are sensitive to PARP inhibition independent of HR repair mechanisms but rather due to suppression of NFκB signaling. This further suggests PARP and NFκB together play a crucial role in carcinogenesis [61] and provides more evidence for the broader utility of PARP inhibitors in cancer therapy.

While these studies are not conclusive evidence that PARP-1 plays a role in tumorigenesis, it is worth considering that PARP-1 regulated signaling may contribute to the initiation or progression of tumors. Interestingly, PARP-1 is highly expressed in a variety of cancers, including breast and hepatocellular carcinoma (HCC), and is correlated with poor prognosis in early breast cancers [62,63,64]. In studies of breast tumors, PARP-1 expression was correlated with higher grade and estrogen receptor (ER) negative status, suggesting that PARP-1 was over-expressed in more aggressive tumor subtypes [63,64]. In the HCC study by Shimizu *et al*., it was found that PARP-1 expression was significantly increased in HCC samples as compared to uninvolved liver tissue, and that PARP-1 expression was also increased in cirrhosis tissues, further implicating PARP-1 in the inflammatory response and in the susceptibility to tumorigenesis [62]. Recently, it was also shown that PARP-1 expression as well as PARylation levels were increased in non-small cell lung cancer cell lines resistant to cisplatin, as compared to their cisplatin-susceptible counterparts, indicating PARP-1 expression and activity may be correlated with tumor resistance to therapy [65]. In addition, PARP-1 and its interaction with the cell cycle control protein p21 (CDKN1A) may also contribute to tumorigenesis and the tumor phenotype. Mechanisms published by Cazzalini *et al*. [66] demonstrate that p21 interacts directly with PARP-1 in the DNA repair process, and knockdown of p21 lead to increased PARP-1 PARylation activity. p21 is often downregulated in cancers due to its regulation by p53 (reviewed in [67]), and based on this study could lead to increased PARP-1 activity. Along these lines, downregulated p21 and subsequent increased PARP-1 activity may provide an additional mechanism through which PARP-1 may play a role in tumorigenesis.

Together, these studies present an interesting perspective on PARP-1 as a tumorigenic factor that is yet to be fully explored. Is chronic activation of PARP-1 in highly oxidative environments responsible for the increased inflammation leading to genome instability and cancer, or are the DNA damages causing an upregulation of PARP-1? One potential mechanism through which PARP-1 and the inflammation-DNA damage feedback loop could be propagated is through the development and attempted repair of oxidative clustered DNA lesions.

## 3. Oxidative Clustered DNA Lesions (OCDLs)

Cells and tissues are constantly exposed to oxidative stresses, which can result in DNA damages that are usually repaired by the base excision repair process, or BER. If the balance between repair mechanisms *versus* ROS oxidation is perturbed, such as in environments where chronic inflammation or highly metabolic tissues reside, accumulation of these types of DNA damage can lead to a phenomenon known as oxidative clustered DNA lesions, or OCDLs.

OCDLs are DNA lesions characterized by two or more bistranded DNA lesions within a 10 base-pair sequence [68]. OCDLs were introduced originally as locally multiply damaged sites (LMDS) in 1981 by Ward *et al*. [69], and have more recently garnered attention due to the possible role of OCDL’s in tumorigenesis and inflammatory disease [70]. The OCDLs can contain a variety of DNA lesions including apurinic-apyramidinic sites, SSBs, and oxidized bases. Endogenous and low levels of exogenous DNA lesions are usually repaired efficiently by BER (which involves PARP-1 activity). However, OCDLs are hypothesized to present more of a challenge to fix, due to the inability of the repair machinery to access the damaged sites within such as small area of DNA [69,71]. Therefore, inefficient repair may lead to the more serious DSB or alternatively, enhanced mutagenicity, with one study showing that 10% of non-double strand break clusters are converted to DSB in NHEJ-deficient CHO cells [72,73]. While lesions greater than 3 bp apart on opposing strands have been shown to result in a DSB upon repair, opposing lesions less than 3 bp apart have been demonstrated to inhibit lesion repair due to the formation of a SSB intermediate which interferes with and/or decreases repair efficiency [74]. *In vitro* work in a bacterial plasmid model has demonstrated that attempted repair of opposing oxidative DNA lesions results in a significantly higher mutation rate within 5,6-dihydrothymine (DHT) and of 8-oxo-7,8-dihydroguanine (8-oxoG) clustered lesions [75]. Additionally, other work in a similar model demonstrated that two closely opposed 8-oxoG lesions could result in higher mutation rates as the BER mechanisms are incapable of effectively repairing both lesions [74,76,77].

### 3.1. OCLDs and Cancer

Endogenous or spontaneous OCDLs occur normally within cells of humans and animals in the range of 200–500 clusters/Gbp [78,79,80]; however, several tumor types have been shown to harbor an increased level of OCDLs [81]. This suggests that there may be correlation between a higher risk of tumorigenesis in tissues and accumulated OCDLs. Additionally, oxidative stress is thought to contribute to tumorigenesis as many chronic inflammatory states lead to increase risk of cancer, such as *H. pylori* infections, hepatitis, and ulcerative colitis [1]. This is due in part to the large production of free radicals in the inflammatory environment leading to increased oxidative DNA damage and genome instability [82]. Decreased clusters were found in cells treated with selenium, a known anti-oxidant, and has been shown to protect against oxidative-induced DNA damage and cancer [79,83]. Functionally, OCDLs may contribute to tumorigenesis as they have been shown to display increased risk for mutagenicity and genetic instability due to the inherent challenges associated with their repair [76,84,85]. Furthermore, IR-induced lesions were shown to persist in mouse tissue 20 weeks post treatment, suggesting these lesions are long lasting and difficult to repair, which could lead to an increased risk of mutagenicity, and tumorigenesis [86].

### 3.2. PARP-1-Friend or Foe of OCDLs?

The potential roles of PARP-1 and OCLDs in tumorigenesis provide a thought-provoking paradigm where in “normal” conditions, ROS induces DNA damage, which is then repaired via the BER process that involves PARP-1 recruitment and enzymatic activity. However, in highly oxidative environments or conditions of chronic inflammation, the sustained upregulation of PARP-1 from the repair process may lead to more inflammation and ROS generation, contributing to the formation of OCDLs. This introduces an interesting question. Is PARP-1 responsible for the increased inflammation leading to genome instability (*i.e.*, through potentiating OCDLs), or are the DNA damages causing an upregulation of PARP-1, and thereby permitting inflammation to influence the cellular environment? This resulting inflammatory feedback loop propagates the phenotype that may be contributing to tumorigenesis. There has been no work to our knowledge that addresses these questions as of yet, but it does present an intriguing area for new research. Additionally, it may provide a feasible avenue for the novel use of PARP-1 inhibitors to prevent cancer outside that of BRCA-associated tumors.

While clinical trials have been conducted for the use of PARP-1 inhibitors in the treatment of cancer, the focus of PARP-1 inhibition was concentrated on its use to obtain synthetic lethality in familial cancers, such as those with a BRCA1/2 mutated phenotypes [87,88,89]. However, this potential connection to OCDLs and inflammation may provide a wider therapeutic application for PARP-1 inhibitors as a potential preventative agent in cancers highly associated with inflammatory phenotypes. By treating with PARP-1 inhibitors, one could reduce the inflammatory process, and thereby also decrease the induction of oxidative induced lesions (like OCDLs) that may lead to cancers.

**Figure 2:**
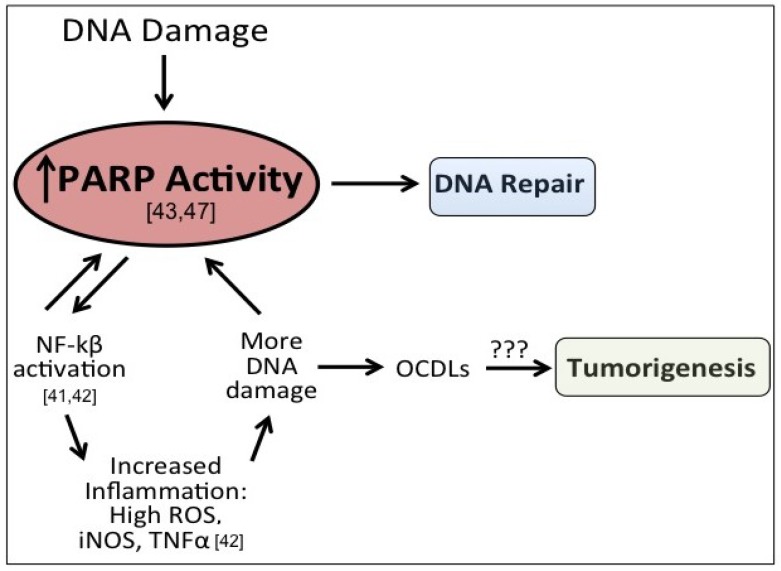
Potential role of elevated PARP-1 in tumorigenesis. After DNA damage, PARP-1 activates DNA repair. However, PARP-1 also acts a co-activator of NFkB signaling, which can propogate inflammatory signaling and lead to more DNA damage, including the formation of oxidatively-clustered DNA lesions (OCDLs). The formation of OCDLs have been shown to be elevated in numerous tumor types. PARP-1 activity could potentially be beneficial or harmful in the repair of ROS-induced DNA lesions.

The principle behind PARP inhibitor treatment of familial cancers deficient in homologous recombination (HR) repair is that by inhibiting the BER pathway in conjunction with endogenous failure of the HR pathway, the cell will be incapable of repairing DNA damage leading to cell death. However, our lab has demonstrated that PARP-1 inhibition also kills HER2+ breast cancer cells despite proficient HR repair, indicating PARP-1 may be an effective treatment for non-familial, sporadic cancers as well as familial cancers [61]. Interestingly, we found that susceptibility of non-familial cancers to PARP-1 inhibition may be through altered NFκB signaling [61]. While the mechanisms behind this are not yet determined, it does provide evidence that the applications of PARP-1 inhibitors in the treatment of cancers may be further extended than originally thought. Therefore, it may be possible to use of PARP-1 inhibitors to not only treat familial cancers, but also to treat and even prevent cancers of non-familial origin, especially cancers associated with chronic inflammation. Chronic PARP-1 activation in tissues undergoing high metabolic activity or tissues subjected to continual ROS-induced stress may be at increased risk for developing cancer driven by PARP-1 signaling, which may be the case for the study in hepatocellular carcinoma mentioned previously which show elevated PARP-1 levels in cirrhotic patients [62]. Together this points to PARP inhibition as an attractive target not only for familial cancers, but also as a treatment, and potentially a preventative agent, for cancers in tissues or organs experiencing chronic ROS-induced damage or high levels of metabolism. A schematic of this potential divergent role of PARP-1 is shown in Figure 2.

## 4. Conclusions

In summary, PARP-1 sits at a critical axis. PARP-1 activity not only assists in repair of ROS-induced DNA lesions, but also can contribute to further damage by promoting inflammation. As a result, upregulated PARP-1 signaling may lead to increased genetic instability and carcinogenesis. Here we have discussed the potential for using PARP-1 inhibition as treatment for not only familial cancers, but also cancers associated with chronic inflammation and high metabolism, which result in high levels of ROS. Therefore, a broader utility of PARP inhibitors likely exists, and further investigation is warranted for future clinical trials in situations of high oxidative stress such that tumor initiation may be prevented.
